# Cognitive Performance Before and Following Habituation to Exercise-Induced Hypohydration of 2 and 4% Body Mass in Physically Active Individuals

**DOI:** 10.3390/nu14050935

**Published:** 2022-02-22

**Authors:** Thomas A. Deshayes, Nicolas Daigle, David Jeker, Martin Lamontagne-Lacasse, Maxime Perreault-Briere, Pascale Claveau, Ivan L. Simoneau, Estelle Chamoux, Eric D. B. Goulet

**Affiliations:** 1 Faculty of Physical Activity Sciences, University of Sherbrooke, Sherbrooke, QC J1K 2R1, Canada; thomas.deshayes@usherbrooke.ca (T.A.D.); nicolas.d.daigle@usherbrooke.ca (N.D.); david.jeker@usherbrooke.ca (D.J.); mllacasse@yahoo.fr (M.L.-L.); maxime.perreault-briere@usherbrooke.ca (M.P.-B.); pascale.claveau2@usherbrooke.ca (P.C.); 2 Research Center on Aging, University of Sherbrooke, Sherbrooke, QC J1H 4C4, Canada; 3 Centre de Recherche et de Formation par Simulation, Cegep of Sherbrooke, Sherbrooke, QC J1E 4K1, Canada; drsimoneau1@gmail.com; 4 Faculty of Arts and Science, Biological Sciences, Bishop’s University, Sherbrooke, QC J1M 1Z7, Canada; echamoux@ubishops.ca

**Keywords:** exercise-induced dehydration, hypohydration, cognitive performance, executive functions

## Abstract

We investigated the effect of repeated exposures to hypohydration upon cognitive performance. In a randomized crossover design, ten physically active adults completed two 4-week training blocks, one where they maintained euhydration (EUH) and the other where they were water-restricted (DEH) during walking/running at 55% $\dot{V}$O_2max_, 40 °C. Three sessions per week were performed: (1) 1 h of exercise, (2) exercise until 2% or (3) 4% of body mass has been lost or replaced. Limited to the first and fourth training week, a 12 min walking/running time-trial was completed following the 2 and 4% exercise bouts. Trail making, the Wisconsin card sort, the Stop signal task, Simple visual reaction time and Corsi block-tapping tests were performed immediately following the time-trials. Body mass loss was maintained < 1% with EUH and reached 2.7 and 4.7% with DEH following the time-trials. Except for a lower percentage of correct responses (% accuracy) during the Wisconsin card sort test (*p* < 0.05) with DEH compared to EUH, no statistically significant decline in cognitive performance was induced by low and moderate levels of hypohydration. Compared to week 1, no statistical differences in cognitive responses were observed after repeated exposures to hypohydration (all *p* > 0.05). From a practical perspective, the gains in cognitive performance following training to DEH were mostly unclear, but under certain circumstances, were greater than when EUH was maintained. Based on the battery of cognitive tests used in the current study, we conclude that whether physically active individuals are habituated or not to its effect, exercise-induced hypohydration of 2 and 4% has, in general, no or unclear impact on cognitive performance immediately following exercise. These results encourage further research in this area.

## 1. Introduction

Heat stress challenges physiological functions [1,2], increases perceived exertion and enhances overall discomfort, the combination of which can lead to an impairment in cognitive performance [3,4]. The extent of impairment appears to be influenced by the severity of heat stress, the duration of exposure and the complexity of the task to be performed [4]. 

Humans have developed coping mechanisms for environmental stressors such as heat stress. Heat acclimation/acclimatization is a strategy that has been shown to mitigate, but not completely prevent, heat-induced cognitive performance decrements [5,6]. Moreover, factors associated with and superimposed on heat stress could also potentially contribute to affecting cognitive performance. 

Sweat losses are accelerated by heat stress exposure and, therefore, if not adequately balanced by fluid intake, will progressively lead to hypohydration. Although conflicting findings have been observed about the impact of hypohydration on cognitive performance [7,8], research shows that the effect, if any, should be relatively small and mainly observed for high-order cognitive processing (i.e., executive function) and motor coordination [7]. Notwithstanding, when present, this physiological state could potentially contribute to worsening the cognitive impairment already associated with heat stress per se. However, it cannot be ruled out that habituation to the impact of hypohydration might contribute to lessen its impact upon cognitive performance.

Few studies provide answers as to whether humans can adapt to repeated exposures to hypohydration. Garrett et al. [9] demonstrated that exposures to low (~2% body mass) hypohydration levels during five consecutive days can improve rather than impair some heat acclimatization-related adaptations. Nonetheless, more recently, these findings were not replicated [10,11,12,13,14]. On the other hand, Fleming and James [15] have shown that four exposures to a low (~2.5% body mass) level of hypohydration attenuate the decrease in exercise performance and perceived exertion. While Garrett et al. [9] and Fleming and James’s [15] studies did not assess cognitive performance, they nevertheless highlight the possibility that the decline in cognitive performance associated with heat stress-induced hypohydration could be somewhat mitigated by the habituation to the repeated effects of hypohydration.

High-order cognitive processing, including executive functions, is essential, especially when tactical and vital decisions must be taken. Indeed, individuals such as military personnel, medical services, firefighters and, in some cases, athletes must maintain a high level of cognitive performance while exposed to hot environments, where access to water is restricted, predisposing them to hypohydration. Therefore, understanding the factors or strategies that could help reduce the effect of hypohydration on cognitive performance under heat-stressful conditions is important from a health and security standpoint [4].

Therefore, the goal of this study was to determine the extent to which low (~2%) and moderate (~4% body mass) hypohydration levels impact cognitive performance following exercise conducted in a hot environment while not habituated to hypohydration, and whether repeated exposures to the effect of hypohydration over 4 weeks moderate the baseline response, compared with a situation where hypohydration is prevented during exercise. It was hypothesized that cognitive performance would be marginally but significantly attenuated by hypohydration while not habituated to its effect, but that after several training sessions with repeated exposures to hypohydration, cognitive performance would be similar whether adequate hydration is being maintained or not during exercise.

## 2. Materials and Methods

### 2.1. Participants

Fifteen (5 women) non-heat-acclimated physically active [16] participants were initially recruited, but ten (2 women) completed the study. Reasons for withdrawal were: knee injury (*n* = 1), fear of exercising while hypohydrated (*n* = 1), health problems (*n* = 1), lack of fitness (*n* = 1) and personal reason (*n* = 1). Women underwent cognitive assessment during the follicular phase of their menstrual cycle (first day of menstruation + the following 13 days, estimated via self-report). Before obtaining their informed written consent, each participant received explanations of the study protocol and associated risks and benefits. The CIUSSS Estrie-CHUS Ethics Committee (#2019-3081) approved all procedures.

### 2.2. Overview of This Study

Both the experimental design of the study and the methods have already been thoroughly detailed elsewhere [17]. Therefore, the following sections will provide only a brief overview of the protocol, and readers are referred to the initial publication for more details. In a randomized and crossover fashion, participants completed two 4-week training blocks (one with fluid access (EUH) and one without fluid access (DEH)) interspersed by a 5-week washout period. Before each training block, baseline measurements and familiarization with the cognitive tests and exercise procedures were performed. For a given training block, participants completed a total of 12 exercise sessions (three per week interspersed by 2 or 3 days). For a given week this included a 1 h exercise bout that always occurred first, followed by two other bouts where participants exercised until 2 or 4% body mass had been lost (DEH training block) or replaced (EUH training block). For the first (pre) and fourth (post) week of each training block (i.e., testing sessions), the 2 and 4% conditions were followed by a 12 min self-paced time-trial. Exercise sessions always occurred at the same time of the day. Cognitive functions were evaluated immediately following exercise during the first and last training week of each block. Participants were restricted to performing physical activity and to consuming sports supplements, respectively, 12 and 24 h prior to their arrival at the laboratory. Moreover, they were required to standardize their food and fluid intake 24 h prior to their arrival at the laboratory. More specifically, participants were asked to keep a dietary log for 24 h prior to the 2 and 4% exercise sessions of the first week of each training block, and then to replicate food and fluid intake before all subsequent 2 and 4% exercise sessions. Participants were also required to consume 250 mL of water 1 h before bedtime and 1 h before their arrival at the laboratory, after which they remained fast. 

### 2.3. Baseline Measurements

After post-void body mass was taken (BX-300 +, Atron Systems, West Caldwell, NJ, USA), height, resting blood pressure, heart rate (Welch Allyn 420 series, Skaneateles Falls, NY, USA) and body composition (DXA, Lunar Prodigy, GE Healthcare, Chicago, IL, USA) were measured. Then, participants underwent a treadmill maximal oxygen consumption ($\dot{V}$O_2max_) test using an expired gas analysis system (Cosmed Quark CPET, Cosmed, Chicago, IL, USA) that had been calibrated with gases of known concentration. 

### 2.4. Familiarization with Cognitive Tests

Overall, participants underwent three familiarization sessions per training block. Two were performed on separate days the week prior to starting the training blocks. During the first, participants came to the laboratory and performed all cognitive tests twice. During the second, participants completed a 1 h walking/light running exercise bout at 55% $\dot{V}$O_2max_, followed by a 12 min self-paced time trial, after which they performed all cognitive tests once. This exercise session was performed while subjects could consume (EUH) or not (DEH) water *ad libitum* (same ambient conditions as during all exercise and testing sessions). The third familiarization session was performed in the fourth week of each training block immediately following the 1 h exercise bout. 

### 2.5. Exercise and Testing Sessions

After they emptied their bladder and collected a urine sample for measurement of urine specific gravity (PAL-10S, Atago, Bellevue, WA, USA) and osmolality (Micro Osmometer, Osmette, Precision Systems Inc., Natick, MA, USA), participants were nude weighted (±50 g, MyWeight HD-300, HBI Technologies, Phoenix, AZ, USA), dressed themselves, installed a heart rate chest electrode (Garmin^TM^, Olathe, KS, USA) and inserted a rectal probe (CoreTemp, HQ, Palmetto, FL, USA). They then entered the environmental chamber (40 ± 0.2 °C and 26 ± 2% relative humidity, wind speed: ~5 km∙h^−1^, solar radiation: 0 W∙m^−2^) and, after an equilibration period of 2 (exercise sessions only) or 10 min (testing sessions only), started walking/light running at an intensity of 55% $\dot{V}$O_2max_. The required intensity was adjusted in the first 10 min of exercise, where O_2_ consumption was continuously measured. Except for the 1 h exercise bout (2 × 30 min interspersed by 3 min rest inside the chamber), participants completed exercise/recovery cycles of 8/2 min until a sweat-induced body mass loss of either 2 or 4% was reached (DEH) or replaced (EUH). Participants were requested to drink water served at room temperature (*ad libitum* intake for the 1 h exercise bout and every 10 min to replace all the sweat and urine losses for the 2 and 4% conditions). Participants could rinse their mouths with water at any time and could pour cold water on their bodies and heads at a rate of 100 mL·h^−1^. After 1 h of exercise and every 30 min thereafter, energy gels (22 g of carbohydrates and 60 mg of sodium, GU Energy gel, Berkeley, CA, USA) were consumed. Heart rate, rectal temperature, perceived exertion (6–20 RPE Borg scale [18]) and thirst (1–11 scale [19]) were measured every 4 min. Only for the testing sessions did participants perform a 12 min time-trial immediately after the fixed-intensity exercise bout. No water was provided during the 12 min time-trial. Immediately following the performance test, participants removed their equipment, toweled themselves, urinated, were weighted and then were transferred to a quiet room (~23 °C) to perform cognitive tests (~10 min from the end of exercise). The hypohydration level (%) was computed as the difference between pre- and post-exercise body mass (post-void) relative to pre-exercise body mass. 

### 2.6. Cognitive Tests

Cognitive tasks were selected to assess executive functions and consisted of the (1) Trail making test (TMT), (2) Wisconsin card sort test (WCST), (3) Stop signal task (SST), (4) Simple visual reaction time test (SVRT), and (5) Corsi block-tapping test (CBT). The tests were always administered in the same order. The cognitive tests (from Millisecond Test Library, Inquisit, Millisecond software, Seattle, WA, USA) were performed in a closed and quiet room using a desktop computer (Windows^®^ 10) with a 19″ diagonal screen (Dell, model 1907FP, Round Rock, TX, USA) and with participants wearing headphones (model MDR-ZX110, Sony Corporation of America, New York, NY, USA) when needed.

#### 2.6.1. Trail Making Test

The TMT is a test of visual attention and task switching that provides information about the speed of processing, visual search speed, scanning, mental flexibility and executive function [20]. It consists of two parts: A (psychomotor speed) and B (switching abilities). In the former, participants are asked to connect in a sequential order 25 points (black circled numbers) presented on a white screen background as quickly as possible using the mouse. In the latter, participants have to connect the points in an alternative order between numbers and letters (e.g., 1, A, 2, B, 3, C, until 13, L). Feedback is automatically provided when an error is made (a yellow halo is presented over the correct circle). A practice block was always performed before the start. Total completion time was taken as the measure of performance. 

#### 2.6.2. Wisconsin Card Sort Test 

The WCST is a test of set-shifting that provides information about the speed of processing and mental flexibility [21]. A card is presented at the top of the white screen background. Four different cards are presented at the bottom of the screen. Participants are asked to classify the cards presented at the top of the screen (2 decks of 24 cards, maximum number of trials = 48) accordingly to 3 different criteria: color of cards’ symbols (green, yellow, blue, red), shape of the symbols (triangle, plus, circle, star), or the number of shapes (1 to 4), using the mouse. The only feedback that is provided to the participants is whether the classification is correct or wrong. An unannounced rule change occurs after 10 consecutive correct responses for a category. Performance was taken as the sum of perseverative errors adjusted relative to the total number of trials (% perseverative errors). Accuracy (% correct responses) was also computed as follows: (total number of correct responses/total number of trials) × 100.

#### 2.6.3. Stop Signal Task 

The SST test measures response inhibition that provides information about impulse control (inhibition ability) [22,23]. Participants are asked to respond to the direction of a white circled arrow (right or left; half the arrows in each trial pointed left) presented on a black screen background by pressing the appropriate key on the keyboard. If an audio tone is heard (no go signal), participants have to inhibit their response. The stop signal response time (SSRT) is an estimate of the response time of the participants to the inhibition of the response, i.e., the time it takes to react to the stop signal by inhibiting the response to the go signal. A longer SSRT indicates more difficulty to stop the go process and vice versa. The SSRT was estimated by the mean method. The test included 64 trials, of which 16 contained a tone. A tracking procedure was used to obtain a broad range of stop signal delays. The latter ranged from 50 to 1150 ms, with adjustment steps of 50 ms depending on the performance. A practice block was always performed before the start. As highlighted by Verbruggen et al. (2008) [24], for participants who inhibit significantly more or less than 50% of the time, the mean method to calculate SSRT should not be used. In such a situation, data were excluded from the analyses. 

#### 2.6.4. Simple Visual Reaction Time Test 

The SVRT test measures the reaction time of participants to respond to a visual target. A fixed black cross is placed on the center of a white screen background. A visual stimulus covers the fixed cross after varying time intervals. The participants have to press the spacebar as soon as the target stimulus appears. Two blocks of 20 trials were completed. The mean reaction time was taken as the measure of performance. 

#### 2.6.5. Corsi Block-Tapping Test 

The CBT measures the visuospatial short-term working memory, which represents the capacity of storage and the temporary management of information [25,26]. Participants are presented with nine blue boxes scattered across the black screen background. The boxes are lighted in yellow in a predetermined order. Afterward, the participants must click on the boxes in the same order they are lit on using the mouse. The length of the sequence starts with 2 and can go up to 9 lit boxes. Participants are provided with 2 trials for each sequence; at least one needs to be completed successfully, otherwise the test ends. A practice block was always performed before the start. The length of the last correctly recalled sequence was measured and taken as the measure of performance.

### 2.7. Statistical Analyses

All statistical analyses were performed using IBM SPSS Statistics software (version 26, New York, NY, USA). Shapiro–Wilk tests were used to analyze data normality. Dependent t-tests or Wilcoxon signed-rank tests were used to compare the characteristics of the participants before each training block. Three factors (hydration*condition*training) were analyzed via ANOVAs and, for variables with missing cases, linear mixed models (with maximum likelihood estimation and scale identity) were used to determine the impact of, and interaction effect between, hydration (EUH vs. DEH), conditions (2 vs. 4%) and training (pre vs. post). Data are presented as mean ± standard deviation (SD). Statistical significance was set at *p* ≤ 0.05. This study reports cognitive-related data collected during a larger scale study whose goals were to determine the physiological, perceptual and performance adaptations associated with repeated exposures to exercise-induced hypohydration. Because cognitive-related data were taken as secondary outcomes, no *a priori* power analysis had been performed. However, to better understand and interpret the results from a practical perspective, data from the second to the third familiarization were used to determine the day-to-day variability for each cognitive test. The practical significance of findings was then determined using the second-generation *p*-value technique, as previously described [27]. 

To obtain the day-to-day variability for each cognitive test, first, the performance data collected during the second familiarization session were subtracted from that obtained during the third familiarization session and, then, the means and associated standard deviations were computed. The smallest worthwhile change in performance for any given cognitive tests was taken as the product of the day-to-day SD × 0.2 (small effect size, [28]). A 95% confidence interval (CI) (i.e., [−0.2 × SD to +0.2 × SD]) was then built. If the statistically derived CI fell completely within this CI, the effect was considered trivial. If it felt completely outside this CI, the data were considered to support a worthwhile negative or positive effect. If the CIs overlapped, the probabilities that the true effect could be declared practically positive, negative or trivial were determined, according to the % overlap. Results were interpreted according to the following descriptors: <1%, almost certainly not; 1–5%, very unlikely; 5–25%, unlikely; 25–75%, possibly; 75–95%, likely; 95–99%, very likely; >99%, almost certainly [29,30]. Worthwhile negative or positive changes in cognitive performance were considered to be outside [−1.08 to 1.08%], [−1.36 to 1.36%], [−5.08 to 5.08 ms], [−0.38 to 0.38] and [−2.33 to 2.33 s], respectively, for the % perseverative errors during the WCST, % accuracy during the WCST, mean reaction time during the SVRT, length of the last correctly recalled sequence during the CBT and the total completion time for the TMT. Such analysis could not be performed for the SST due to computer-related problems during the third familiarization session.

## 3. Results

### 3.1. Participants Characteristics at Baseline and Prior to Starting the Second Training Block

At baseline, mean age, height, body mass, body mass index, maximal oxygen consumption, maximal heart rate, fat-free mass and fat mass were, respectively, 23 ± 5 years, 176 ± 8 cm, 71.6 ± 11.8 kg, 23.1 ± 2.9 kg·m^−2^, 55 ± 7 mL·kg^−1^·min^−1^, 193 ± 7 beats·min^−1^, 82.4 ± 6.5% and 14.0 ± 6.6%. A comparison of the baseline values shows that body mass (∆_block2-block1_ = −0.4 ± 1.2 kg), body mass index (∆ = −0.15 ± 0.4 kg·m^−2^), $\dot{V}$O_2max_ (∆ = −1.0 ± 2.6 mL∙kg^−1^∙min^−1^), fat-free mass (∆ = −0.15 ± 1.8%) and fat mass (∆ = 0.2 ± 1.6%) were not significantly different before the first and second training block (all *p* > 0.05). 

### 3.2. Baseline Hydration Status

Participants started all testing sessions in a well-hydrated state. Indeed, as demonstrated in Table 1, plasma natremia and urine specific gravity and osmolality were within the physiological range and did not differ among trials. However, there was a hydration (both *p* < 0.01) effect for pre-exercise urine specific gravity and urine osmolality, with no other main or interaction effects (all *p* > 0.05). Moreover, pre-exercise body mass (maximum difference across all trials: 0.8 kg, corresponding to < 1% of body mass variation) and plasma osmolality (average of 288.5 ± 5.0 mOsm·kg^−1^) did not differ among trials and were suggestive of adequate hydration. 

### 3.3. Exercise Duration

Total exercise duration (fixed-intensity exercise bout + 12 min self-paced time-trial) reached 75.8 ± 9.5 and 141.4 ± 16.9 min with the 2 and the 4% conditions, respectively (*p* = 0.0001), without a difference between EUH and DEH (*p* = 0.45) or a hydration*condition effect (*p* = 0.08). Total exercise duration decreased through training, in both conditions, and was significantly lower during the second testing session (−7 ± 4 min, *p* = 0.0001). No hydration*condition*training effect was observed (*p* = 0.15). The overall mean exercise intensity during the fixed-intensity exercise bout was 56 ± 2% $\dot{V}$O_2max_ (all *p* > 0.05).

### 3.4. Fluid Balance during Exercise

**Figure 1:**
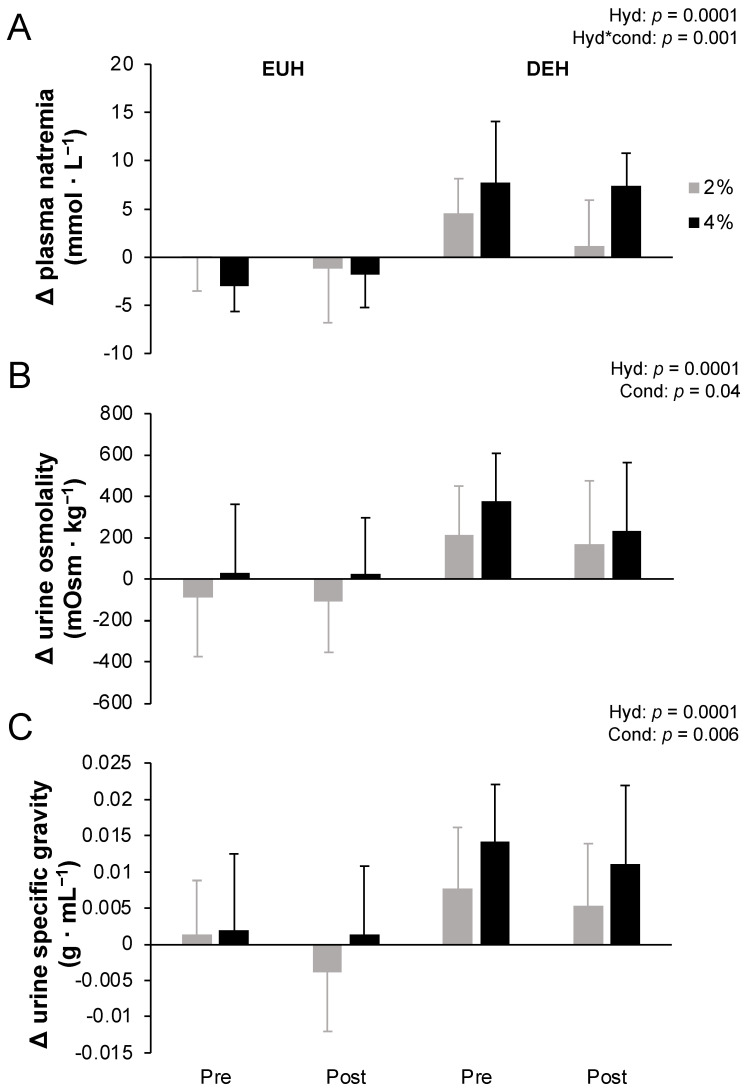
Changes from pre- to post-exercise in plasma natremia measured at the end of the fixed-intensity exercise period (**A**), and urine osmolality (**B**) and urine specific gravity (**C**) measured at the end of the 12 min time-trial while replacing or not 2 or 4% body mass losses, pre- and post-training. Values are means ± SD. Δ = difference. EUH = euhydrated; DEH = dehydrated; Cond = condition (2 vs. 4%); Hyd = hydration (EUH vs. DEH). Only the significant effects are reported.

At the end of the 12 min time-trial, body mass losses remained <1% for EUH (0.8 ± 0.2%) and reached 2.7 ± 0.2% and 4.7 ± 0.2% for DEH with the 2 and the 4% conditions, respectively. Figure 1 depicts changes in plasma natremia from pre- to post-exercise measured at the end of the fixed-intensity exercise period (A), and urine osmolality (B) and urine specific gravity (C) measured at the end of the 12 min time-trial while replacing or not 2 or 4% body mass losses, pre- and post-training. Accordingly, plasma natremia increased more with DEH than EUH (*p* = 0.0001), with a hydration*condition (*p* = 0.001) but no other effects (all *p* > 0.05). Post hoc analyses revealed that a hydration effect was present for both conditions (both *p* < 0.01), but that a condition effect was only present with DEH (*p* = 0.0008) but not EUH (*p* = 0.17). Similarly, at the end of the 12 min self-paced time-trial, urine osmolality (250 ± 234 vs. −53 ± 224 mOsm·kg^−1^, respectively) and specific gravity (0.010 ± 0.008 vs. 0.000 ± 0.007 g·mL^−1^, respectively) increased more with DEH than EUH (both *p* = 0.0001), with a condition effect (*p* = 0.04 and 0.006) but no other main or interaction effects (all *p* > 0.05).

### 3.5. End of Performance Physiological and Perceptual Responses

**Figure 2:**
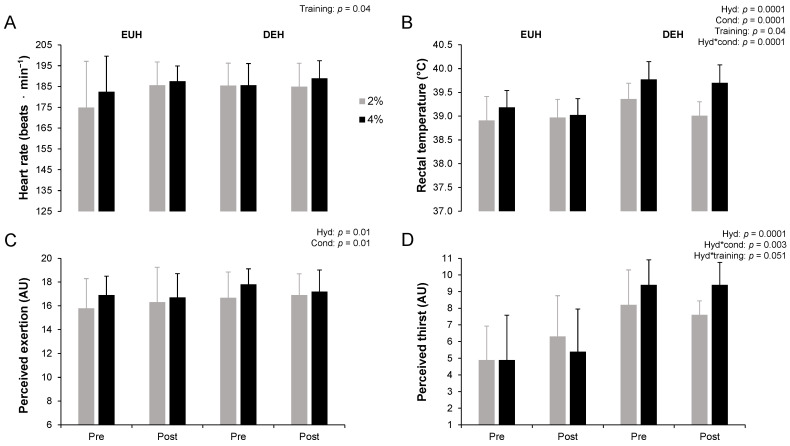
Heart rate (**A**), rectal temperature (**B**), perceived exertion (**C**) and perceived thirst (**D**) at the end of the 12 min time-trial while replacing or not 2 or 4% body mass losses, pre- and post-training. Values are means ± SD. EUH = euhydrated; DEH = dehydrated; Cond = condition (2 vs. 4%); Hyd = hydration (EUH vs. DEH); Training (pre vs. post); AU: arbitrary units. Only the significant effects are reported.

Physiological and perceptual responses have been published elsewhere [17]. The overall mean exercise heart rate maintained during the 12 min time-trial was 179 ± 14 beats·min^−1^, representing 93 ± 7% of maximal heart rate. The end of performance rectal temperature, perceived exertion and perceived thirst (all *p* < 0.05), but not heart rate (*p* = 0.11), were significantly higher with DEH compared to EUH (Figure 2). Hydration*condition effects were observed for rectal temperature and perceived thirst. Post hoc analyses revealed that a condition effect was present with EUH (*p* = 0.03) and DEH (*p* = 0.0001) for rectal temperature, but only with DEH for perceived thirst (*p* = 0.001). Values were higher with DEH compared to EUH in the 2% condition (rectal temperature: +0.2 ± 0.4 °C, *p* = 0.005; perceived thirst: +2 ± 3, *p* = 0.0001) as well as in the 4% condition (rectal temperature: +0.6 ± 0.3 °C; perceived thirst: +4 ± 2, both *p* = 0.0001). The end of performance heart rate (+4 ± 13 beats·min^−1^) and rectal temperature (−0.1 ± 0.3 °C), respectively, increased and decreased through training (both *p* = 0.04), similarly between EUH and DEH, as suggested by the absence of a hydration*training effect (*p* = 0.16 and 0.17, respectively). No hydration*training effect was observed for perceived thirst (*p* = 0.051). However, post hoc analyses revealed that thirst perception was higher with DEH compared to EUH pre- and post-training (*p* < 0.05). Interestingly, perceived thirst at the end of performance increased through training with EUH (+1 ± 2, *p* = 0.048), but remained stable with DEH (−0.3 ± 2, *p* = 0.50). No other effects were observed (all *p* > 0.05).

### 3.6. Cognitive Performance

Overall, cognitive performance was not affected by low and moderate levels of hypohydration. Compared to the baseline, no differences were observed after repeated exposures to hypohydration. The mean duration to complete the five cognitive tests remained constant throughout trials (23 ± 1 min, all *p* > 0.05).

**Figure 3:**
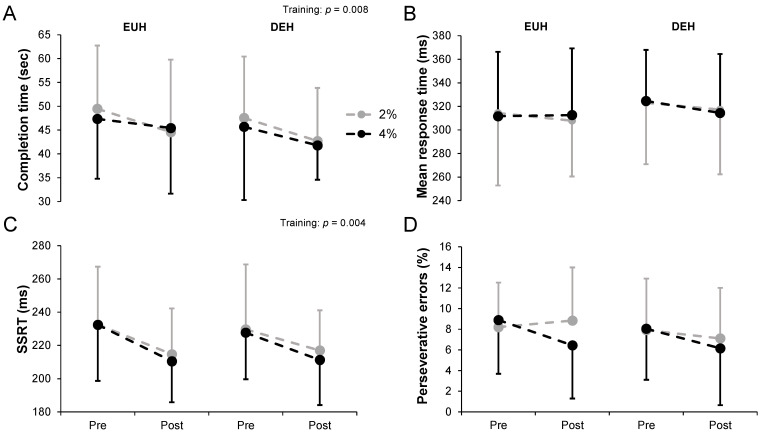
Changes in the total time to complete the Trail making test (**A**), mean response time during the Simple visual reaction time (**B**), mean stop signal response time during the Stop signal task (**C**) and perseverative errors during the Wisconsin card sort test (**D**) while replacing or not 2 or 4% body mass losses, pre- and post-training. Values are means ± SD. EUH = euhydrated; DEH = dehydrated; Training (pre vs. post); SSRT: Stop signal response time. Only the significant effects are reported.

Figure 3 shows the changes in cognitive performance for the TMT (A), SVRT (B), SST (C) and WCST (D) tests while replacing or not 2 or 4% body mass losses, pre- and post-training. On average, participants completed the TMT with a mean duration time of 46.71 ± 13.35 s with EUH and 44.39 ± 11.67 s with DEH (*p* = 0.25), without condition (*p* = 0.73) or hydration*condition (*p* = 0.97) effects. The average time to complete the TMT decreased with training (*p* = 0.008), without hydration*training (*p* = 0.56) or condition*training (*p* = 0.74) effects. No hydration*condition*training effect was observed (*p* = 0.54). While no hydration effect was observed overall, interpreting the data from part A and B depicts a different conclusion. No main interaction effects (all *p* > 0.05) were observed in part A (training, *p* = 0.08). However, the time to complete part B was significantly lower with DEH compared to EUH (22.32 ± 6.74 vs. 25.05 ± 8.41 sec, *p* = 0.02), with training (*p* = 0.007) but no other effects (all *p* > 0.05). For the SVRT, a slightly but non-significantly longer mean reaction time was observed with DEH compared to EUH (319.8 ± 48.5 vs. 311.6 ± 53.2 ms, *p* = 0.09). No other main or interaction effects were observed (all *p* > 0.05). The mean SSRT during the SST slightly decreased with training (*p* = 0.004) from 230.5 ± 32.8 (pre) to 213.4 ± 24.7 ms (post), without hydration (*p* = 0.70), condition (*p* = 0.56) or any interaction effects (all *p* > 0.05). 

No main or interaction effects (all *p* > 0.05) were observed for percentage of perseverative errors during the WCST (training, *p* = 0.07). However, hydration (*p* = 0.001) and hydration*training (*p* = 0.03), but no other main or interaction effects (all *p* > 0.05), were observed for accuracy (% correct responses). Post hoc analyses revealed that a hydration effect was only observed in post- (EUH: 79 ± 18 vs. DEH: 72 ± 26%, *p* = 0.004) but not in pre-training (EUH: 76 ± 16 vs. DEH: 75 ± 20%, *p* = 0.38), without a training effect either with EUH or with DEH (both *p* > 0.05). For the CBT, the lengths of the last correctly recalled sequence were 7.3 ± 0.7 (EUH pre), 7.2 ± 0.9 (DEH pre), 7.3 ± 0.7 (EUH post) and 7.3 ± 1.1 (DEH post) with the 2% condition. These values reached 7.3 ± 0.8, 7.4 ± 1.1, 7.3 ± 0.9 and 7.4 ± 0.8, respectively, with the 4% condition. No main and no interaction effects were observed (all *p* > 0.05).

Table 2 reports the observations derived from the second-generation *p*-value analyses. Regarding the training effect (i.e., change in cognitive performance from pre- to post- training with 2 or 4% hypohydration), a 64% chance of worthwhile effect with DEH compared to EUH was observed in the 4% condition for the SVRT. Similarly, a 54% (2% condition) and 57% (4% condition) chance of worthwhile effect with DEH compared to EUH was observed for the WCST and the TMT, respectively. In other words, such results highlight that, for these tests, the gains in cognitive performance following training to hypohydration have been observed to be possibly greater than when EUH was maintained. Conversely, regarding % accuracy during the WCST, a practical advantage with EUH has been observed, with improved % accuracy from pre- to post-training with EUH training (2%:69%; 4%:100%), while it decreased with DEH. Other effects were trivial or unclear.

## 4. Discussion

The aims of this study were to determine (1) the impact of low and moderate hypohydration levels on cognitive performance while not habituated to hypohydration and (2) whether repeated exposures to the effect of hypohydration moderate the baseline response. While previous studies were specifically designed to verify whether repeated exposures to hypohydration could lessen its negative impact upon endurance performance or physiological functions, this is the first one to investigate whether habituation to hypohydration could mitigate the potential cognitive impairment associated with hypohydration immediately following exercise. The main findings of this study were: in physically active individuals, immediately following exercise and compared with euhydration, (1) low and moderate exercise-induced hypohydration do not impair cognitive performance, (2) repeated exposure to exercise-induced hypohydration over a 4-week period did not change these outcomes from a statistical perspective and (3) from a practical perspective, the impact of repeated exposure to exercise-induced hypohydration is mainly unclear, highlighting that more studies in this area are necessary. 

Contrary to our first hypothesis, our results indicate that exercise-induced hypohydration ranging from 2 to 4% of body mass does not impair cognitive performance following exercise when unhabituated to being exposed to its multifaceted effects. Therefore, the current findings do not support the reported claim that fluid loss ≥2% body mass [7,31] negatively affects cognitive performance and, more specifically, the executive functions, which play a critical role in the ability of a person to navigate easily through everyday life situations. We are not the first study to observe an absence of a statistically significant effect of hypohydration on cognitive performance. Indeed, several previous studies [32,33], including a recent meta-analysis [8], have come to the conclusion that exercise-induced hypohydration does not lower cognitive performance, compared with when euhydration is maintained during exercise. This observation holds true irrespective of the cognitive domain being assessed [8], or when body mass loss reaches up to 4–5% [34,35]. 

It is frequently argued that symptoms associated with hypohydration, including but not limited to thirst, mental fatigue and impaired mood [35,36,37], may potentially act as a distraction and, thus, reduce cognitive performance [38]. In this regard, an hyperosmotic stimulus [39], and the associated triggering of the thirst response [40,41], have been previously shown to alter mood, exacerbate mental fatigue and decrease cognitive performance. We did not observe such a cognitive decrement with hypohydration in spite of the higher, and expected, natremia level and thermal (i.e., rectal temperature), cardiovascular (i.e., heart rate) and perceptual (i.e., thirst and perceived exertion) strains. Furthermore, no cognitive performance decrement was observed, despite an average rectal temperature >39 °C at the end of the time-trial with DEH, a threshold at which hyperthermia-associated cognitive impairments typically occur [42]. Indeed, Schmit et al. [42] reported an inverted U relationship between hyperthermia and cognitive impairment, with a threshold fixed at ~39 °C for cognitive impairments. 

Our second hypothesis was that repeated exposures to low and moderate exercise-induced hypohydration would moderate, in a positive manner, the baseline response. More specifically, we proposed that there would be no difference in cognitive efficiency between EUH and DEH at post-testing, implying that the degree of improvement in cognitive performance through training would be more important with DEH than EUH. Because no hypohydration-related cognitive decrements were observed at baseline between EUH and DEH following exercise, while participants were not familiar with the effect of hypohydration, unfortunately, our results prevent us from answering the question of whether or not the brain can develop while submitted to repeated bouts of hypohydration, specific adaptations to reestablish cognitive functions to a euhydrated-associated baseline level. That being said, we could have observed that the cumulative effect of repeated exposures to low and moderate hypohydration induces mental fatigue over time and, as a result, lessen cognitive performance at post-testing. However, such a trend was not observed, and repeated exposures to hypohydration did not worsen the baseline response. In other words, no cognitive impairments were observed with repeated exposures to low and moderate hypohydration beyond exercise-induced EUH. This observation is particularly important for firefighters [43], military personnel [44] or individuals that are exposed to hot environments with limited access to hydration over long or consecutive shifts/days. In fact, it suggests that limited access to fluids for 3 days per week over a period of several weeks is unlikely to be detrimental to cognitive performance, compared with the maintenance of euhydration during exercise. We previously reported that the impact of repeated exposures to hypohydration upon endurance performance remains quite similar through training [17]. Such an observation is congruent with the status quo observed in cognitive performance through training in the present study. 

In addition to the classical statistical approach, we performed a practical analysis using the second-generation *p*-value. The latter aimed to provide a clearer perspective on the practical implication of the present findings. Regarding the changes in cognitive performance from pre- to post- training, at 2% hypohydration, the overall effect seems uncertain since three of the five results were unclear and a contradiction was observed between the other two (Table 2). At 4% hypohydration, a practical advantage was observed with DEH for two of the five results, with one in advantage to EUH, one trivial and one unclear. Thus, the overall effect seems also unclear. Taken together, our results do not demonstrate that attempting to train people to tolerate hypohydration could bring gains in cognitive performance lower than when EUH is maintained, but that in certain circumstances the gains in cognitive performance following training to hypohydration could be greater than when EUH is maintained. However, such a constate remains uncertain, and encourages more studies in this area.

Various methodological reasons may explain why we observed statistically insignificant or unclear effects of hypohydration on cognition. Participants completed several familiarization trials with the cognitive tests. Moreover, the tests were always performed in the same sequence. It is not impossible that this combination of factors may have contributed to our inability to observe an effect of hypohydration on cognitive performance. Another explication may be related to the overall difficulty of the tasks that participants had to complete, or to the overall task time, which may not have been long enough to pick up mental fatigue. Indeed, it has been recognized that at least 30 min of engagement in difficult and demanding cognitive tasks are necessary to induce mental fatigue, and thus to identify a possible cognitive impairment [45]. Exercise per se is known to enhance cognitive performance. Indeed, it has previously been demonstrated that aerobic exercise has a small but beneficial effect on cognitive performance, and more specially on executive functions, attention and information processing [46,47]. This effect may last for up to 20 min after exercise completion [46]. Because ~10 min separated the end of the exercise period from the cognitive assessment phase, it cannot be excluded that exercise may have counteracted any potential deleterious impact of hypohydration. However, the residual impact of exercise on cognitive functions appears to be more pronounced in children and the elderly compared to adults [48]. Moreover, Wittbrodt and Millard-Stafford [7] and others [49,50] have concluded that the method used to achieve hypohydration (i.e., exercise alone, exercise + heat stress, water restriction) does not influence how hypohydration impacts cognitive performance. There is the possibility that physically active individuals can inherently possess a good tolerance to the impact of hypohydration upon cognitive performance compared with sedentary individuals. To this effect, Wittbrodt and Millard-Stafford [7] showed a negative impact of hypohydration on cognitive performance that is nearly twofold lower in highly fit individuals compared to their recreationally fit counterparts. Or, alternately, these individuals may show more resilience to the influence of water loss on cognitive abilities. In this regard, Kempton et al. [51] observed a higher neuronal activity during the execution of a cognitive task in hypohydrated adolescents. Had we worked with a population with a lower fitness level, it cannot be excluded that a different outcome would have been observed. It cannot be excluded that our inability to systematically observe interaction effects between hydration and training may be due to a lack of sensitivity of some of the tests we used or a lack of statistical power. However, it must be borne in mind that, collectively, the battery of cognitive tests used was associated with several significant differences, including a hydration*training effect for the WCST (% accuracy), as well as practical effects according to the second-generation *p*-value analyses. Not only does the latter approach to data interpretation provide a clearer perspective on the practical implication of the present findings, but also, and importantly, it improves the robustness of our findings. Irrespective of the reasons why we could not witness an impact of hypohydration on cognitive functions, our observations highlight the fact that cognitive and physical performance may be dissimilarly deranged by hypohydration [17]. Nonetheless, such results are explorative, and more research on this topic is necessary before we can make robust conclusions.

## 5. Conclusions

From a statistical perspective, whether a physically active individual has never been or has repeatedly been exposed to low (2%) and moderate (4%) exercise-induced hypohydration under hot ambient conditions, based on the battery of cognitive tests we used, our results indicate that, immediately following exercise, it does not impact his or her ability to provide a cognitive performance that is similar to when these body losses are fully replaced through water consumption. From a practical perspective, although the gains in cognitive performance following training to DEH were mostly unclear, under certain circumstances, they were greater than when euhydration was maintained. Further studies in this research area are required.

## Figures and Tables

**Table 1:** Plasma natremia, urine osmolality and specific gravity data before each testing session with euhydration and dehydration through conditions and training.

Variables	First Testing Session		Second Testing Session	
	2% Condition	4% Condition	2% Condition	4% Condition
	Euhydrated			
Plasma natremia (mmol·L^−1^)	136.4 ± 3.0	137.8 ± 1.7	135.8 ± 4.4	137.8 ± 1.9
Urine osmolality (mOsm·kg^−1^)	573 ± 374	589 ± 374	675 ± 376	503 ± 363
Urine specific gravity (g·mL^−1^)	1.014 ± 0.010	1.016 ± 0.011	1.019 ± 0.009	1.014 ± 0.010
	Dehydrated			
Plasma natremia (mmol·L^−1^)	137.4 ± 2.9	138.4 ± 4.3	137.4 ± 2.5	136.6 ± 3.8
Urine osmolality (mOsm·kg^−1^) *	441 ± 312	339 ± 302	373 ± 324	346 ± 361
Urine specific gravity (g·mL^−1^) *	1.012 ± 0.008	1.011 ± 0.009	1.010 ± 0.009	1.009 ± 0.009

Values are means ± SD. *: *p* ≤ 0.05 between EUH and DEH (hydration effect).

**Table 2:** Practical analysis of the changes in cognitive performance from pre- to post-training with the 2 or the 4% condition.

Cognitive Test	Condition	Conclusion *	Interpretation
WCST (% perseverative errors)	2%	Practically positive: 54% Practically negative: 23% Trivial: 23%	**DEH possibly beneficial**
	4%	Practically positive: 29% Practically negative: 43% Trivial: 28%	**Unclear**
WCST (% accuracy)	2%	Practically positive: 9% Practically negative: 69% Trivial: 21%	**EUH possibly beneficial**
	4%	Practically positive: 0% Practically negative: 100% Trivial: 0%	**EUH is almost certainly beneficial**
SVRT (mean reaction time in ms)	2%	Practically positive: 42% Practically negative: 44% Trivial: 14%	**Unclear**
	4%	Practically positive: 64% Practically negative: 11% Trivial: 24%	**DEH possibly beneficial**
CBT (length of the last correctly recalled sequence)	2%	Practically positive: 36% Practically negative: 26% Trivial: 39%	**Unclear**
	4%	Practically positive: 22% Practically negative: 22% Trivial: 57%	**DEH possibly trivial effect**
TMT (total completion time in sec)	2%	Practically positive: 32% Practically negative: 33% Trivial: 35%	**Unclear**
	4%	Practically positive: 57% Practically negative: 23% Trivial: 20%	**DEH possibly beneficial**

EUH = euhydrated; DEH = dehydrated; * = compared with EUH; WCST: Wisconsin card sort test; SVRT: Simple visual reaction time test; CBT: Corsi block-tapping test; TMT: Trail making test.

## Data Availability

The data will be made available from the corresponding author upon reasonable request.

## References

[1] González-Alonso J., Crandall C.G., Johnson J.M. (2008). The cardiovascular challenge of exercising in the heat. J. Physiol..

[2] Périard J.D., Cramer M.N., Chapman P.G., Caillaud C., Thompson M.W. (2011). Cardiovascular strain impairs prolonged self-paced exercise in the heat. Exp. Physiol..

[3] Hancock P.A., Vasmatzidis I. (2003). Effects of heat stress on cognitive performance: The current state of knowledge. Int. J. Hyperth..

[4] Martin K., McLeod E., Périard J., Rattray B., Keegan R., Pyne D.B. (2019). The impact of environmental stress on cognitive performance: A systematic review. Hum. Factors.

[5] Racinais S., Wilson M.G., Gaoua N., Périard J.D. (2017). Heat acclimation has a protective effect on the central but not peripheral nervous system. J. Appl. Physiol..

[6] Radakovic S.S., Maric J., Surbatovic M., Radjen S., Stefanova E., Stankovic N., Filipovic N. (2007). Effects of acclimation on cognitive performance in soldiers during exertional heat stress. Mil. Med..

[7] Wittbrodt M.T., Millard-Stafford M. (2018). Dehydration impairs cognitive performance: A meta-analysis. Med. Sci. Sports Exerc..

[8] Goodman S.P., Moreland A.T., Marino F.E. (2019). The effect of active hypohydration on cognitive function: A systematic review and meta-analysis. Physiol. Behav..

[9] Garrett A., Goosens N., Rehrer N., Patterson M., Harrison J., Sammut I., Cotter J. (2014). Short-term heat acclimation is effective and may be enhanced rather than impaired by dehydration. Am. J. Hum. Biol..

[10] Haroutounian A., Amorim F.T., Astorino T.A., Khodiguian N., Curtiss K.M., Matthews A.R., Estrada M.J., Fennel Z., McKenna Z., Nava R. (2021). Change in Exercise Performance and Markers of Acute Kidney Injury Following Heat Acclimation with Permissive Dehydration. Nutrients.

[11] Travers G., Nichols D., Riding N., González-Alonso J., Périard J.D. (2020). Heat acclimation with controlled heart rate: Influence of hydration status. Med. Sci. Sports Exerc..

[12] Pethick W.A., Murray H.J., McFadyen P., Brodie R., Gaul C.A., Stellingwerff T. (2019). Effects of hydration status during heat acclimation on plasma volume and performance. Scand. J. Med. Sci. Sports.

[13] Schleh M.W., Ruby B.C., Dumke C.L. (2018). Short term heat acclimation reduces heat stress, but is not augmented by dehydration. J. Therm. Biol..

[14] Neal R.A., Corbett J., Massey H.C., Tipton M.J. (2016). Effect of short-term heat acclimation with permissive dehydration on thermoregulation and temperate exercise performance. Scand. J. Med. Sci. Sports.

[15] Fleming J., James L.J. (2014). Repeated familiarisation with hypohydration attenuates the performance decrement caused by hypohydration during treadmill running. Appl. Physiol. Nutr. Metab..

[16] McKay A.K., Stellingwerff T., Smith E.S., Martin D.T., Mujika I., Goosey-Tolfrey V.L., Sheppard J., Burke L.M. (2022). Defining Training and Performance Caliber: A Participant Classification Framework. Int. J. Sports Physiol. Perform..

[17] Deshayes T.A., Daigle N., Jeker D., Lamontagne-Lacasse M., Perreault-Briere M., Claveau P., Simoneau I.L., Chamoux E., Goulet E.D.B. (2021). Impact of repeated acute exposures to low and moderate exercise-induced hypohydration on physiological and subjective responses and endurance performance. Nutrients.

[18] Borg G.A. (1982). Psychophysical bases of perceived exertion. Med. Sci. Sports Exerc..

[19] Goulet E.D., Rousseau S.F., Lamboley C.R., Plante G.E., Dionne I.J. (2008). Pre-exercise hyperhydration delays dehydration and improves endurance capacity during 2 h of cycling in a temperate climate. J. Physiol. Anthropol..

[20] Tombaugh T.N. (2004). Trail Making Test A and B: Normative data stratified by age and education. Arch. Clin. Neuropsychol..

[21] Heaton R.K., Chelune G.J., Talley J.L., Kay G.G., Curtis G., Curtiss G., Heaton R., Chelune G., Talley J., Kay G. (1993). Wisconsin Card Sorting Test (WCST)—Manual Revised and Expanded.

[22] Verbruggen F., Aron A.R., Band G.P., Beste C., Bissett P.G., Brockett A.T., Brown J.W., Chamberlain S.R., Chambers C.D., Colonius H. (2019). A consensus guide to capturing the ability to inhibit actions and impulsive behaviors in the stop-signal task. eLife.

[23] Verbruggen F., Logan G.D. (2008). Response inhibition in the stop-signal paradigm. Trends Cogn. Sci..

[24] Verbruggen F., Logan G.D., Stevens M.A. (2008). STOP-IT: Windows executable software for the stop-signal paradigm. Behav. Res. Methods.

[25] Berch D.B., Krikorian R., Huha E.M. (1998). The Corsi block-tapping task: Methodological and theoretical considerations. Brain Cogn..

[26] Kessels R.P., Van Zandvoort M.J., Postma A., Kappelle L.J., De Haan E.H. (2000). The Corsi block-tapping task: Standardization and normative data. Appl. Neuropsychol..

[27] Deshayes T.A., Jeker D., Goulet E.D.B. (2020). Impact of Pre-exercise Hypohydration on Aerobic Exercise Performance, Peak Oxygen Consumption and Oxygen Consumption at Lactate Threshold: A Systematic Review with Meta-analysis. Sports Med..

[28] Cohen J. (1988). Statistical Power Analysis for the Behavioral Sciences.

[29] Batterham A.M., Hopkins W.G. (2019). The Problems with “The Problem with ‘Magnitude-Based Inference”. Med. Sci. Sports Exerc..

[30] Hopkins W.G., Marshall S.W., Batterham A.M., Hanin J. (2009). Progressive statistics for studies in sports medicine and exercise science. Med. Sci. Sports Exerc..

[31] Sawka M.N., Burke L.M., Eichner E.R., Maughan R.J., Montain S.J., Stachenfeld N.S. (2007). American College of Sports Medicine position stand. Exercise and fluid replacement. Med. Sci. Sports Exerc..

[32] Wittbrodt M.T., Millard-Stafford M., Sherman R.A., Cheatham C.C. (2015). Fluid Replacement Attenuates Physiological Strain Resulting from Mild Hypohydration without Impacting Cognitive Performance. Int. J. Sport Nutr. Exerc. Metab..

[33] Goodman S.P., Moreland A.T., Marino F.E. (2019). Maintaining Euhydration Preserves Cognitive Performance, But Is Not Superior to Hypohydration. J. Cogn. Enhanc..

[34] Van Den Heuvel A.M., Haberley B.J., Hoyle D.J., Taylor N.A., Croft R.J. (2017). The independent influences of heat strain and dehydration upon cognition. Eur. J. Appl. Physiol..

[35] Ely B.R., Sollanek K.J., Cheuvront S.N., Lieberman H.R., Kenefick R.W. (2013). Hypohydration and acute thermal stress affect mood state but not cognition or dynamic postural balance. Eur. J. Appl. Physiol..

[36] Ganio M.S., Armstrong L.E., Casa D.J., McDermott B.P., Lee E.C., Yamamoto L.M., Marzano S., Lopez R.M., Jimenez L., Le Bellego L. (2011). Mild dehydration impairs cognitive performance and mood of men. Br. J. Nutr..

[37] Armstrong L.E., Ganio M.S., Casa D.J., Lee E.C., McDermott B.P., Klau J.E., Jimenez L., Le Bellego L., Chevillotte E., Lieherman H.R. (2012). Mild Dehydration Affects Mood in Healthy Young Women. J. Nutr..

[38] Cheuvront S., Kenefick R. (2014). Dehydration: Physiology, assessment, and performance effects. Compr. Physiol..

[39] Suh H., Lieberman H.R., Jansen L.T., Colburn A.T., Adams J., Seal A.D., Butts C.L., Kirkland T.M., Melander O., Vanhaecke T. (2021). Cellular dehydration acutely degrades mood mainly in women: A counterbalanced, crossover trial. Br. J. Nutr..

[40] Goodman S.P.J., Marino F.E. (2021). Thirst perception exacerbates objective mental fatigue. Neuropsychologia.

[41] Edmonds C.J., Crombie R., Gardner M.R. (2013). Subjective thirst moderates changes in speed of responding associated with water consumption. Front. Hum. Neurosci..

[42] Schmit C., Hausswirth C., Le Meur Y., Duffield R. (2017). Cognitive functioning and heat strain: Performance responses and protective strategies. Sports Med..

[43] Cvirn M.A., Dorrian J., Smith B.P., Vincent G.E., Jay S.M., Roach G.D., Sargent C., Larsen B., Aisbett B., Ferguson S.A. (2019). The effects of hydration on cognitive performance during a simulated wildfire suppression shift in temperate and hot conditions. Appl. Ergon..

[44] Lieberman H.R., Bathalon G.P., Falco C.M., Kramer F.M., Morgan C.A., Niro P. (2005). Severe decrements in cognition function and mood induced by sleep loss, heat, dehydration, and undernutrition during simulated combat. Biol. Psychiatry.

[45] Pageaux B., Lepers R. (2018). The effects of mental fatigue on sport-related performance. Prog. Brain Res..

[46] Chang Y.-K., Labban J.D., Gapin J.I., Etnier J.L. (2012). The effects of acute exercise on cognitive performance: A meta-analysis. Brain Res..

[47] Lambourne K., Tomporowski P. (2010). The effect of exercise-induced arousal on cognitive task performance: A meta-regression analysis. Brain Res..

[48] Ludyga S., Gerber M., Brand S., Holsboer-Trachsler E., Pühse U. (2016). Acute effects of moderate aerobic exercise on specific aspects of executive function in different age and fitness groups: A meta-analysis. Psychophysiology.

[49] Cian C., Koulmann N., Barraud P., Raphel C., Jimenez C., Melin B. (2000). Influences of variations in body hydration on cognitive function: Effect of hyperhydration, heat stress, and exercise-induced dehydration. J. Psychophysiol..

[50] Cian C., Barraud P.A., Melin B., Raphel C. (2001). Effects of fluid ingestion on cognitive function after heat stress or exercise-induced dehydration. Int. J. Psychophysiol..

[51] Kempton M.J., Ettinger U., Foster R., Williams S.C., Calvert G.A., Hampshire A., Zelaya F.O., O’Gorman R.L., McMorris T., Owen A.M. (2011). Dehydration affects brain structure and function in healthy adolescents. Hum. Brain Mapp..

